# 

**Published:** 1886-09

**Authors:** 


### HALL'S JOURNAL of HEALTH

ESTABLISHED 1854

CONTENTS.

Vol. 33 No. 9

| | PAGE. |
|---|---|
| Special | 249 |
| Natural Sleep | 249 |
| The Healing Power | 252 |
| Left-Handed Reformation | 254 |
| Nature the Great Teacher | 256 |
| Medical Quacks | 253 |
| Verification of a Dream | 259 |
| The Throat | 260 |
| A Word of Caution to Woolen Mill Operatives | 261 |
| Twelve Ways of Injuring the Health | 262 |
| Some Ancient Anecdotes | 263 |
| Health Cure | 264 |
| A Good Work | 264 |
| Writer's Cramp | 265 |
| A Prescription | 266 |
| Home Department | 267 |
| Miscellaneous Items | 269 |

INDEX TO ADVERTISEMENTS OF HALF A PAGE AND OVER.

| | PAGE. |
|---|---|
| Lactopeptine | 272 |
| Castoria | 272 |
| Fellows' Hypo-Phosphites | I |
| Lactated Food | II |
| Celerina | III |
| Horsford's Acid Phosphate | IV |
| Caulocorea | IV |
| Bovinine | V |
| Vaginal Injecting and Suction Syringe | VII |
| Medicated Suppositories | VIII |
| Hall's Journal of Health—Nature's Assistants | 2d page cover |
| Perforated Paper | 3d " " |
| Saved from the Knife | 4th " " |

